# Clinical audit of Ankylos implant abutment fractures for a ten-year period in practice

**DOI:** 10.1038/s41415-024-8009-0

**Published:** 2025-06-13

**Authors:** Sally Rayment, Mark Packer, Brian J. Millar

**Affiliations:** 168030623180074470734 Postgraduate Tutor, Faculty of Dentistry, Oral and Craniofacial Sciences, King´s College London, United Kingdom; 661775972299872457348 Faculty of Dentistry, Oral and Craniofacial Sciences, King´s College London, United Kingdom

## Abstract

**Introduction** Abutment fracture is a clinical problem which can lead to loss of an implant.

**Aim** To compare abutment fracture rate in the authors' practice against published data and identify any risk factors.

**Methods** The published literature on the subject was reviewed and a retrospective ten-year clinical audit was conducted to determine the rate of implant abutment fracture for all single-unit, Ankylos, screw-retained implants restored on a titanium base.

**Results** The abutment fracture rate obtained from the literature was 2.2%, with a specific Ankylos rate of 1.8%. The overall abutment fracture rate in practice during the audited ten-year period was 0.6%, with the following contributing factors identified: male patients (62%), first molar site (84% were at first molar sites), implant restoration opposing a natural tooth (89%), implant bound by another tooth/implant (64%), implant functioning for approximately six years before fracture and a gonial angle of <120°.

**Conclusion** The fracture rate from the audit was 1.6% lower than that obtained from the literature search and 1.2% lower than that set specifically for Ankylos implants in the published literature. Risk factors were identified which should be considered when planning and consenting cases.

## Key points

- Titanium bases form both the base and the connection to the titanium implant, allowing for integration into the digital workflow and good aesthetics while achieving higher fracture resistance than ceramic abutments.
- Titanium bases, however, can fracture, with the main risk factors identified in this audit as being: male patients - 62%; implant replacing first molar - 84%; implant opposing natural tooth - 89%; implant functioning in the mouth for approximately six years.
- This ten-year clinical audit looked at 17,027 single-unit, screw-retained Ankylos implants gleaning a titanium base fracture rate of 0.6%, compared with the published average sample size of 1,207 and rate of 1.8%.

## Introduction

Dental implants can be an excellent solution for the partially or fully edentate; they are considered by some to be the best available option for these type of patients.^1^ Over the years, the criteria for success of dental implants have changed from simply desiring osseointegration and survival in the mouth^2^ to expecting life-like restorations, healthy and natural-looking peri-implant soft tissues, good aesthetics, and patient satisfaction.^3^ Survival rates can be misleading as implants that are retained despite disease being present, rather than being removed, can create a grey area for measurement of success.^4^

With the increasing popularity of dental implants and higher numbers of implants now being placed, the dental profession is now more aware of the complications that can occur. Patients need to be informed of these potential outcomes as part of the consent process and any identifiable risk factors that may lead to complications should be discussed.

The possible complications can be broken down into two broad categories: surgical and prosthodontic.^5^ The prosthodontic category can be further broken down as follows:^6^

- Biological (peri-implant mucositis/peri-implantitis)
- Mechanical (screw-loosening/fracture, chipped/fractured restoration, fixture fracture, retention loss, abutment fracture)
- Aesthetic/phonetic (loss of interdental papilla/e, mucosal recession, poor restoration contour, shade mismatch).

Peri-implantitis has several/many causative factors, including those related to biotype, position of the implant, oral hygiene and risk factors, as well as prosthodontic factors, such as excess luting cement.

Within the mechanical complications subgroup, abutment fracture has been reported to happen infrequently - according to some studies, as little as 0.35%,^7^ with other studies reporting higher frequencies of 3.3-5.5%.^8^^,^^9^ Abutment fracture occurs when occlusal force is transmitted from the superstructure to the abutment due to occlusal loading, as well as when unloading forces occur and metal fatigues.

The implant-abutment connection within the Ankylos (DentsplySirona) system has a conical internal connection, with the conical interface as a Morse taper (5.7°). This allows the platform switch and abutment to be narrower than that of the implant diameter, ensuring the implant-abutment gap is away from the implant shoulder, lending itself to superior bone preservation.^10^

Titanium-base abutments were designed to overcome aesthetic problems of titanium and the low fracture resistance of ceramic abutments, as these combine titanium and ceramic components, with the titanium component forming the base and connection to the titanium implant.^11^ Titanium bases can be associated with increased risk of crown debond, and as we will find, fracture. Screw fracture is rare with titanium bases and the crown debond is often the ‘weak link' in the system, allowing the crown to debond before risking abutment or screw fracture.

The titanium base allows for computer-aided design/computer-assisted manufacturing and permits a more economical production of the implant restoration. However, there is currently limited evidence to demonstrate how these will fare in the long-term.^12^

One of the main risk factors for abutment fracture is that of excessive occlusal loading of the implant prosthesis, either by being incorrectly restored, or by the unmanaged parafunctional habits of the patient. If implant crowns are restored in supra-occlusion, damage to the componentry can occur over time as either porcelain fracture, crown decementation, or abutment fracture.

For patients with ongoing unmanaged bruxism (characterised by worsening tooth surface loss) or unmanaged clenching habits allowing high forces to be put through the dentition, the risk of abutment fracture is greater.^13^ Poor prosthesis design, such as too long a span of crown, can also contribute to abutment fracture, as the occlusal forces are some distance away from the centre of the implant, creating an almost cantilever effect.

**Fig. 1:**
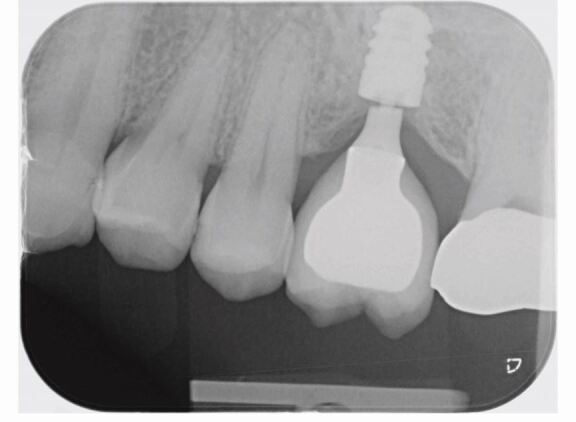
Periapical radiograph showing how a fractured abutment presents once the screw is accessed and undone

Abutment fracture usually presents to a patient initially as a seemingly loose crown. Upon presentation to the dentist, abutment fracture can be diagnosed by undoing the screw and taking a periapical radiograph, which will usually show a distinct radiolucency where the fracture line is (Fig. 1). Abutment fractures can be preceded by crowns with chipped/fractured porcelain, or a debonded crown that has been re-bonded without any occlusal adjustment.

**Fig. 2:**
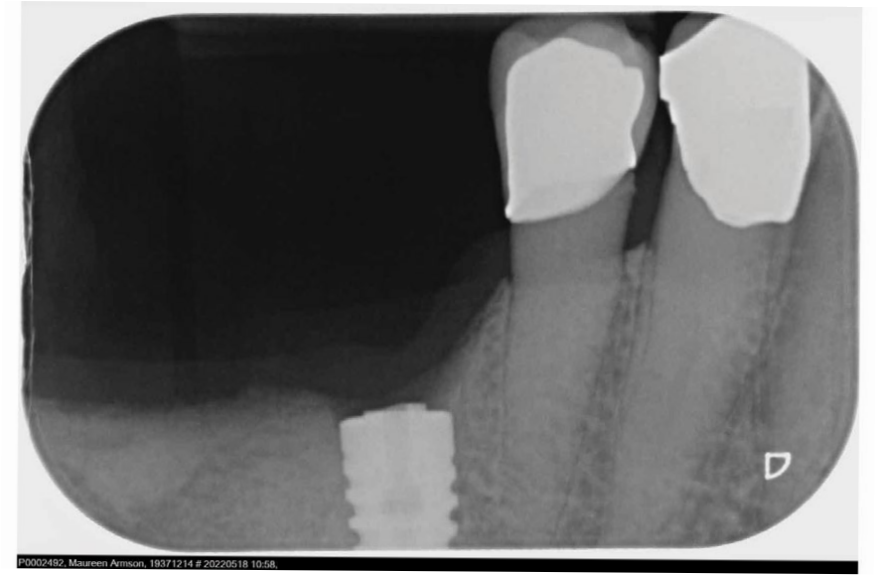
Periapical radiograph showing the apical portion of the fractured abutment still in the implant once the loose coronal portion has been removed

Management of the fractured abutment is usually unpleasant for the patient as the loose coronal portion needs to be forcefully removed, subsequently followed by careful retrieval of the apical portion of the abutment. Figure 2 shows a periapical radiograph of the apical portion of the abutment in the 46 implant that is yet to be retrieved.

If this part cannot be retrieved successfully without damaging the implant, the implant has effectively failed, as it can no longer be re-restored.

**Fig. 3:**
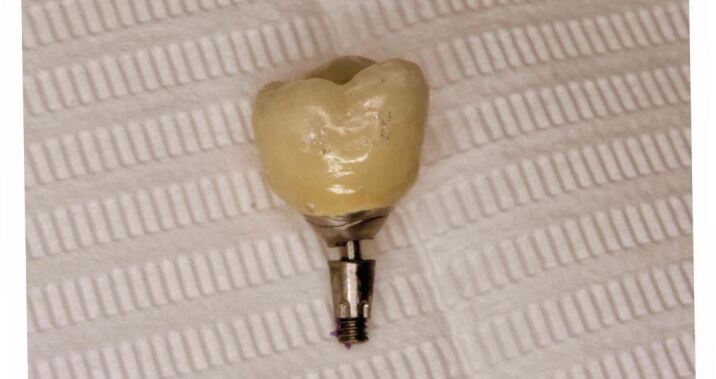
Photograph of crown and fractured abutment successfully removed in its entirety

Occasionally, the entire abutment and screw come out, removing the loose coronal portion (Fig. 3). This simplifies the management as a new titanium base can then be re-bonded to the crown if it is not damaged, or completely remade if it is. The main objective when faced with a fractured abutment is to remove the abutment as carefully as possible so as not to damage the internal surface of the implant, allowing it to be restored.

The Ankylos abutment screw has the threads laser welded to it, allowing the screw to be mobile but not removable. Excessive torque can lead to fracture of the screw. The design is such that if this occurs, the screw shaft separates from the thread, and once the abutment is removed from the implant, the thread can be removed with the DentsplySirona Ankylos unscrew instrument. If the screw fractures above the thread ring the shaft, retrieval is more challenging but is possible with the correct approach and equipment.

Miwa *et al.*^14^ found an inverse correlation between the gonial angle (GOA) on the orthopantomograms (OPGs) and the maximum occlusal force recorded at the first molar sites for their sample population. This could be an interesting tool to use when assessing patients for implants to replace their first molars as a predictor for possible abutment fracture.

The aim of this study was to audit the fractured implant abutments in practice over a ten-year period, then compare the results to the fracture rate obtained from a review of the published literature. The management of the risk factors can be highlighted and implemented to help reduce the fracture rate and any patterns that emerge can be used to help prevent future abutment fracture.

## Methods

### Literature search

**Fig. 4:**
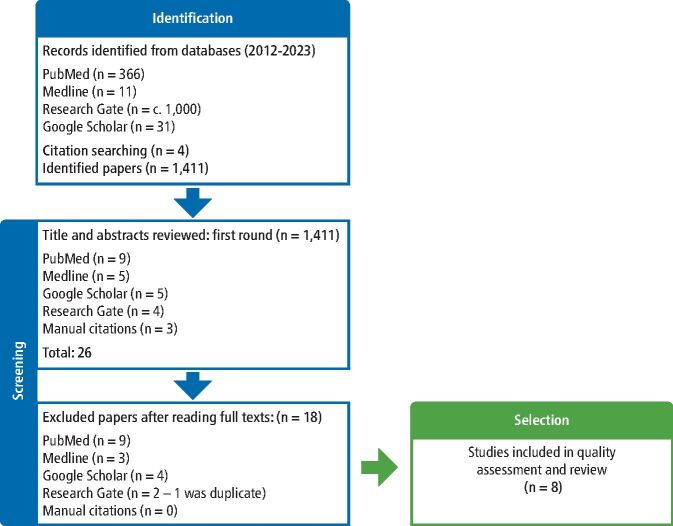
Flowchart of fractured abutment rate literature search

To determine the abutment fracture rate from the literature to compare the outcome of the practice clinical audit, it was apparent that while undertaking a preliminary review of the literature for ‘titanium base abutments', a search of the relevant literature should be broadened to encompass all types of implant abutments, not just ‘titanium bases'. The term on initial investigation was too specific and narrowed the search results down considerably, as well as missing relevant publications. Therefore, a decision was made to include all titanium abutments in the search, with the aim to exclude studies with zirconia/ceramic abutments, which behave in a completely different manner to their metal equivalents. Figure 4 shows how the search was conducted.

Four electronic databases (PubMed, Medline, ResearchGate and Google Scholar) were searched using the following combinations of key words:

- Dental implants AND implant abutment fracture OR titanium base fracture OR mechanical implant complications; Dental implant AND Ankylos abutment fracture OR titanium base fracture.

Papers were included if they satisfied the research criteria with the additional inclusion requirements:

- Publications 2012-present, helping to ensure they are contemporary and relevant to current clinical practice
- Written in English
- Multiple reports published from the same study were considered, with the most comprehensive and up-to-date publication being selected for review.

The reference lists of the selected papers were then manually searched and any relevant journal articles that satisfied the search criteria were added to the identified publications. For eligibility criteria, an adaptation of the evidence-based research strategy ‘PICO' was adopted.

PICO stands for:

- P - patient/population
- I - intervention
- C - comparison - what is the main alternative (control)?
- O - outcome - what is trying to be accomplished/measured/improved?

For this scenario, there is no formal intervention being compared. The format adopted was therefore PECO, with the E substituting the I as an ‘exposure' as opposed to an intervention:

- Population - patients with a single tooth fixed prosthesis (restored implant)
- Exposure - patients with a fractured titanium base
- Comparison - patients without a fractured titanium base
- Outcome - reported failure rate of titanium bases and reasons for failure.

The following data items were extracted from each study:

- Author/s
- Year of publication
- Research type and setting
- Sample size at baseline and follow-up
- Baseline characteristics of trial participants/samples (age, sex, abutment type)
- Inclusion and exclusion criteria
- Fracture abutment rates versus comparison group of non-fractured abutments (if given)
- Follow-up period
- Outcome (reported failure rate of titanium bases and reasons for failure).

For quality of the evidence, the resulting papers were appraised using the Oxford Centre for Evidence-based Medicine's levels of evidence (Table 1). A quality score was then allocated to each study to give a numeric quantification and rank the evidence.^15^

**Table 1:** Quality of the studies included in the review

**Question**	**Di Francesco25**	**Shim and Yang19**	**Yang et al.20**	**Joda et al.26**	**Jin et al.21**	**Murakami et al., 202016**	**Yi et al.22**	**Yi et al.9**
1. Is question/objective/aim sufficiently described?	1	1	1	1	1	1	1	1
2. Is study design evident and appropriate?	1	1	1	1	1	1	1	1
3. Is method of subject/control group selection fully described and appropriate?	1	1	1	1	1	1	1	1
4. Is the setting of study /study population clearly defined?	1	1	1	1		1	1	1
5. Subject and comparison group characteristics (age and sex) sufficiently described and clearly defined?	1	1	1	1	1	1	1	1
6. Was random allocation used?	N/A	N/A	N/A	N/A	N/A	N/A	N/A	N/A
7. Was it stated how subjects were attained?	1	1	1	1	1	1	1	1
8. Were the inclusion criteria stated?	1	1	1	1	1	1	1	1
9. Were the exclusion criteria stated?	1	1	1			1	1	1
10. Was the sample size for each group stated?	1	1	1	1	1	1	1	1
11. Was the treatment fully described for the intervention group?	1		1	1	1	1	1	1
12. Was the treatment fully described for the control group?	1		1			1	1	1
13. Were groups compared on any variables?	1	1				1	1	
14. Were the outcome measures clearly defined?	1	1	1	1	1	1	1	1
15. Were the outcome measures tested for validity and reliability?		1	1	1	1	1		1
16. Were the outcome assessors blinded?								
17. Were the participants blinded?								
18. Was there more than one examiner?		1	1					
19. Were examiners calibrated?		1	1					
20. Was the statistical analysis carried out?	1	1	1	1	1	1	1	1
21. Was the statistical significance defined?		1	1	1	1	1	1	1
22. Was the follow up stated?	1	1	1	1	1	1	1	1
23. Was the follow up greater than 80%?	1		1					1
24. Was the conclusion stated?	1	1		1	1	1	1	1
**Quality score**	**17**	**18**	**20**	**15**	**14**	**18**	**17**	**18**

Relevant papers were scarce, largely due to the limited amount or research studies looking specifically at fractured abutments, rather than reporting as a finding in a multiple outcome study. The identification of papers reporting the fracture of titanium bases was even more challenging, partly due to the fact they are a relatively new treatment modality. The fractured abutment percentages from these included papers were analysed and an average deduced to compare with the dental practice audit outcome (Table 2).

**Table 2:** Included studies and abutment fracture rates reported

**Authors**	**Research Type**	**Target Population**	**Setting**	**Exposure**	**Results given**	**Follow-up**	**Level of evidence**	**Quality of evidence**
Di Francesco *et al*.^25^	Retrospective clinical study	172 Astra Tech OsseoSpeed internal hexagon implants (Dentsply Sirona) placed in 85 patients with a follow-up between January 2009 and January 2019	Multidisciplinary Department of Medical, Surgical, and Dental Sciences of Campania University, Luigi Vanvitelli	Patients with a fractured titanium base	2.9% abutment fracture rate	10 years	2b	17/24
Shim and Yang^19^	Retrospective clinical cohort study	257 patients with Ankylos implants placed between 2005-2012	Dental clinics at an urban university hospital	Patients with a fractured abutment	2.2% abutment fracture rate	Mean follow-up of implants was 63.5 ± 16.0 months (5 years)	2b	18/24
Yang *et al.*^20^	Retrospective clinical cohort study	495 patients treated with 945 Ankylos implant system between 2012-2020	Dental department of the Zhejiang Provincial People's Hospital	Patients with a fractured titanium base	1.38% abutment fracture rate	9 years	2b	20/24
Joda *et al.*^26^	Prospective clinical trial	44 patients restored with 50 screw-retained monolithic implant crowns on Straumann implants	University of Bern	Patients with any implant complications	0% abutment fracture rate	2 years	2b	15/24
Jin *et al.*^21^	Retrospective clinical cohort study	5,563 patients treated with 10,231 Straumann implants, and 1,260 patients treated with 2307 Ankylos implants between 2012−2018	Zhejiang University School of Medicine, China	Patients with a fractured abutment	2.2% for the Ankylos group	8 years	2b	14/24
Murakhami *et al.*^16^	Retrospective clinical cohort study	1,126 Ankylos implants in 430 patients, between January 2008 - December 2009	Oral Implant Clinic, Nihon University Hospital at Matsudo	Patients with a fractured abutment	1.6% abutment fracture rate	10 years	2b	18/24
Yi et al.^22^	Retrospective clinical study	428 patients with 898 internal conical connection Warantec implants	Seoul National University Dental Hospital Institutional Review Board	Patients with fractured abutment	11.4% abutment fracture rate	14 years	2b	17/24
Yi *et al.*^9^	Retrospective clinical study	406 patients with 1,289 Warantec implants - total 490 internal connection implants, over 12-year period from 2002-14	Seoul National University Dental Hospital	Patients with a fractured abutment	8.2% abutment fracture rate (40/490 internal connection implants)	12 years	2b	18/24

## Clinical audit

The practice being audited, which specialises in the placement of dental implants, has been placing Ankylos implants since 2007. These implants have been fitted by eight different implant surgeons, five of whom are qualified maxillofacial surgeons, two are both specialist prosthodontists and oral surgeons, and one is a specialist oral surgeon. The implants were restored by seven clinicians in the practice, three of whom are specialist prosthodontists and four who are non-specialist restorative clinicians. Some of the implants were also restored by external referring general dental practitioners, who received the relevant Ankylos restorative training at the practice before performing the restoration procedure(s).

Reports were generated from the practice's Software of Excellence software, showing all the emergency codes that could be relevant to a patient attending with a fractured abutment. These included:

- Fractured abutment
- De-bonded crown from titanium base
- Fractured porcelain
- Loose crown
- Pain
- Emergency.

Records were then assessed to see exactly how many patients presented with a fractured titanium base on an Ankylos implant between 01/01/2012 and 31/12/2022, as it was around this time that the practice moved away from cement-retention to screw-retention.

Each record was searched to ensure that no fractured abutments were missed from the audit. The data were then input into an Excel spreadsheet.

The GOA was determined by measuring the angle formed between the inferior border of the mandible and the posterior border of the ramus. Studies have shown that this measurement is on a par with the GOAs measured from lateral cephalograms.^16^^,^^17^

The records revealed that 105 patients had a fractured abutment associated with an Ankylos screw-retained implant crown. The following variables were recorded:

1. Sex of patient (male/female)
2. Age of patient when abutment fractured (years)
3. Site of implant in mouth
4. Implant length/mm
5. Implant diameter/mm
6. Period of time implant had been in mouth before fractured abutment (months)
7. Whether the abutment was successfully removed and implant restored
8. Whether the opposing tooth was natural/implant/edentulous space/denture
9. If the implant was a terminal tooth or bound by another tooth/teeth/implant
10. Whether the GOA (measured from OPG) was less than or greater than 120°.

The decision to look at these ten variables in the audit was made after reading the included papers, then looking at what were measured and recorded in their retrospective studies. These variables have been implicated as causative factors for abutment fracture and were therefore recorded in the audit.

The practice began placing Ankylos implants in 2007 but did not introduce screw-retained crowns until 2011. Therefore, to know a true fracture rate for the abutments in scope, all implants placed and restored with screw-retained restorations on a titanium base since 2011 have been analysed. Auditing one specific brand of implant helps to reduce any fracture rate variances attributed to differing implant design and implant-abutment connection.^9^

The total number of Ankylos implants restored as screw-retained implant crowns placed was determined (less those that had failed to integrate pre-loading). Data were then collected to see how many of these were molars and of these, how many were fixed-fixed bridge abutments and full-arch-fixed bridge abutments. This allowed deviation of the figure for total implants placed and restored as single molar units.

The data were then analysed to see how many of the fractured abutments belonged to the first molars and in turn, the fracture rate for each site (16, 26, 46 and 36). Other data fields were looked at, such as whether the restored implant was the most distal in the arch or bound by another tooth/implant and whether the implant crown was opposing a natural tooth or implant.

The overall abutment fracture rate percentage could then be calculated and statistical analyses applied to compare this percentage to the fracture rate determined from the literature research to answer the questions that underpin the purpose of the study.

## Results

### Results from literature search

Relevant papers regarding abutment fracture were challenging to locate, as this does not appear to be a topic that has been greatly researched to date. That said, many papers exist regarding ceramic/zirconia abutments, but these papers have been excluded as previously mentioned. Metal abutment fracture incident rates were difficult to come by and titanium-base fracture rates even more so, possibly due to the fact they are relatively new to the market. Figure 4 depicts the process of selecting the papers to include in the study and how the decision to reach the final eight articles was reached.

#### Quality of included studies

As can be seen from Table 2, a total of eight papers were selected to establish the fracture rate, with their quality ranging from 14/24 to 20/24, giving an average quality score of 17/24, a median of 17.5 and an interquartile range of 15.5−18.

#### Follow-up periods of the included studies

Table 2 shows that the follow-up period for the retrospective clinical studies ranged from 2-14 years. The mean being 8.75 years with a median of 9.5 years, and an interquartile range of 5.75−11.5.

The follow-up periods were also looked at for the four included studies which looked at Ankylos implants: the range was 5-10 years, the mean was 8 years, the median was 8.5 years and the interquartile range was 5.75−9.75 years. The mean follow-up period for all eight studies and the four Ankylos studies were similar (8.75 and 8 years, respectively).

The eight included articles all looked at cohorts of varying numbers of implants in their study periods. This ranged from 50-12,538 implants, giving a mean of 2,083.6, with an interquartile range of 241.5−1,080.75, and a median of 694. Due to the outlier of 12,538, the median is the most useful number here.

The next area of focus was to look at the number of implants included in the four Ankylos studies in the hope that this would provide a more useful mean due to the narrower range of implant numbers included. This gave a range of 450-2,307 implants, a mean of 1,207, an interquartile range of 573.75−2,011.75 and a median of 1,035.5.

From the abutment fracture rates reported in the retrospective clinical studies, there is a range of incidence from 0-11.4%, giving an average fracture rate of 3.7%, with an interquartile range of 1.4−6.9 and a median and mode of 2.2%.

The range of abutment fracture rate percentages from the studies relating specifically to Ankylos implants is 1.4-2.2%, with a mean of 1.8%, an interquartile range of 1.4−2.2%, and a median of 1.9%.

Due to the broad range of fracture rate incidence across the eight studies, the average may not be the most reliable statistic to determine the published fracture rate. Therefore, if the values from all eight studies are to be used, the median of 2.2% should be used to compare the practice audit to. However, it may be more sensible to use the data from the four studies looking at the Ankylos implants, which gives an average implant abutment fracture rate of 1.8%.

### Results from clinical audit

#### Differences in abutment fracture rate between women and men

Within the defined ten-year period of the audit (01/01/2012 to 31/12/2022), there have been 105 cases of titanium-base fractures on Ankylos implants that have been restored as screw-retained crowns on titanium bases. Of this, 40 patients were female (38.1%) and 65 were male (61.9%). This correlates with previous studies which have shown that men are more likely to experience abutment fracture due to larger maximum generated occlusal.^14^^,^^18^ This phenomenon will be further analysed with the GOA measurements.

#### Ages of patients with fractured abutments

The average age was 61 years at time of abutment fracture, with an interquartile range of 54−68 years, a median of 60 years and a range of 35-85 years old (with one outlier at 91 years). Three of the included studies gave average ages for the fractured abutment patients of 52.5, 54 and 49 years, respectively,^19^^,^^20^^,^^21^ resulting in a meta-analysis average of 52 years. This is nine years younger than the average age in the audit.

#### Site in mouth

The notation of the tooth that the implant was replacing was also collected. The most prevalent sites for implant fractures were the first molar regions, with 36 being the most frequent (32/105), followed by 46 (24/105), 16 (16/105) and 26 (15/105), giving a total abutment fracture percentage for the first molars of 83%. In total, there were 8/105 fractured abutments that were premolars (7.5%) and 10/105 that were second molars (9.5%).

**Fig. 5:**
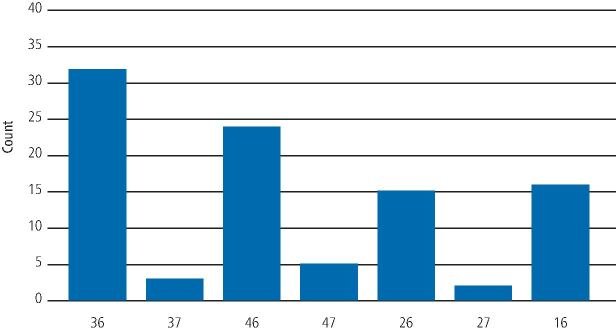
Frequency of abutment fractures according to molar site in the mouth

Due to the high frequency of molars, especially the first molars being involved with abutment fractures, the premolars were then excluded from the audit, leaving a total of 97 fractured abutments to analyse. The frequencies of abutment fractures for molar sites are depicted in Figure 5.

There have been a greater number of first molar implants involved in abutment fracture. As there were no 17 implants with fractured abutments, it was therefore then decided to look at the first molars explicitly to see if any patterns emerged.

The total number of first molar implants with fractured abutments were 87, with 36 having 32 (36.4%), 46 having 24 (27.3%), 16 having 16 (18.2%) and 26 having 15 (17%).

As it appeared the rate for the lowers was significantly greater than that for the uppers, a chi-square test was performed to compare abutment fracture rates for the upper and lower first molars. The chi-square test was performed using Microsoft Excel, testing the null hypothesis that the proportion of implants with fractured abutments was equal whether they were on an upper first molar or a lower first molar versus the alternative hypothesis that the proportion was not equal. The assumptions underlying the chi-square test were satisfied.

There were 1,916 single-unit upper first molars in the audit, of which 31 (1.6%) had a fractured abutment, with 1,885 (98%) without a fractured abutment. There were 2,793 single-unit lower first molars in the audit, of which 56 (2%) had a fractured abutment, with 2,737 (98%) without a fractured abutment.

The hypothesis test showed an insignificant result (p >0.05; p = 0.8) and the null hypothesis can therefore not be rejected. The conclusion from this is that the proportion of implants with fractured abutments is equal whether they are an upper or lower first molar. This contrasts with the findings in the Jin *et al.*, 2022 study,^21^ which found a statistical difference between maxillary and mandibular implants where p = 0.03.

Looking at the differences between male and female patients, the initial data of fractured abutment rate between them seemed significantly different. Therefore, a chi-square test was performed to see if there was a difference in first molar abutment fracture rates between men and women. This tested the null hypothesis that the proportion of first molar implants with fractured abutments was equal whether they occurred in male or female patients, versus the alternative hypothesis that the proportion was not equal. The assumptions underlying the chi-square test were satisfied.

There were 2,030 single-unit first molars belonging to male patients, of which 52 (2.6%) had a fractured abutment, with 1,978 (97.4%) without a fractured abutment. There were 2,679 single-unit lower first molars belonging to female patients, of which 35 (1.3%) had a fractured abutment, with 2,644 (98.7%) without a fractured abutment.

The hypothesis test showed a significant result (p <0.05; p = 0.0015) and the null hypothesis can therefore be rejected. The conclusion from this test is that the proportion of implants with fractured abutments between male and female patients is different. This correlates with the findings in five of the eight included studies used to determine the abutment fracture rate.

Shim and Yang found no statistical difference between male and female patients with respect to abutment fracture rate.^19^ However, the other four studies showed a statistically significant difference in p value between male and female patients, which correlates with the findings in this audit.

#### Period of time implants were functioning in mouth before abutment fracture

The period of time the implants were functioning in the mouth before abutment fracture was also recorded. The range of months that implants were functional in the mouth before abutment fracture was 6-165 (with an outlier of 178), with a mean of 74.3, an interquartile range of 45-94.5 and a median of 72.

#### Fractured abutment retrieval and implant re-restoration

Except for four cases, all the apical portions of the fractured abutments were successfully retrieved without damage, allowing the implant to be successfully re-restored. Of the four patients that were not successfully retrieved, two did not return and the other two had to have the implant removed as their retrieval was not possible. This means that 96% of the implants with fractured abutments were saved and re-restored successfully.

#### Opposed by natural tooth or implant: is it the terminal tooth?

Another finding in the data relates to whether the implants with the fractured abutments were opposing a natural tooth or an implant. The data revealed that 89% of the implants with fractured abutments were opposing a natural tooth and 11% were opposing an implant. None of the implants in this audit were opposed by an edentulous space or denture. Additionally, 64% of the fractured abutments belonged to an implant that was bound by a tooth/implant distal to it, while 36% belonged to an implant that was the most distal in the arch.

A chi-square test was also performed for these last two variables of whether the opposing tooth was a natural tooth or an implant and whether the implant with the fractured abutment was a terminal tooth or not. This showed that, of the molar implants with fractured abutments, 56 were opposing a natural tooth and were bound by another tooth/implant in the arch, 30 were opposing a natural tooth and were the terminal tooth in the arch, and the remaining eleven were all opposed by an implant, with an almost even split between being the most distal tooth or bound by another tooth/implant. To see if this difference was statistically significant, a chi-square test was conducted.

A chi-square test was performed in SPSS (version 27) to test the null hypothesis that the proportion of implants with fractured abutments was equal, whether they were the terminal tooth in the arch or not, and if they were opposed by a natural tooth or an implant, versus the alternative hypothesis that the proportion was not equal. The assumptions underlying the chi-square test were satisfied.

There were 11 implants with fractured abutments opposing an implant, of which six (54.5%) were not a terminal toot and five (45.5%) were a terminal tooth. There were 86 implants with fractured abutments opposing a natural tooth, of which 56 (65%) were not a terminal tooth and 30 (35%) were a terminal tooth.

The hypothesis test showed an insignificant result (p >0.05; p = 0.255) and the null hypothesis can therefore not be rejected. The conclusion from this is that the proportion of implants with fractured abutments is equal, whether they are the terminal tooth or not, and if they are opposed by natural tooth or implant.

#### Gonial angle

The GOA on the OPGs of patients with fractured first molar abutments was measured and recorded as either being less than or greater than 120°, as this is the critical angle quoted in the literature. Three cases had to be excluded due to the absence of an OPG for their case.

In total, 77% of patients with first molar fractured abutments had GOA <120° and 23% had GOA>120°. This shows that most patients with fractured abutments had a GOA of less than 120°. This agrees with the evidence in the literature that there is great occlusal force in patients with a GOA of less than 120°.

This audit has also found significantly more male patients with fractured abutments. Therefore, a chi-square test was performed in SPSS (version 29) to test the null hypothesis that the proportion of first molar implants with fractured abutments occurring in male or female patients was equal whether the GOA was less than or greater than 120°, versus the alternative hypothesis that the proportion was not equal. The assumptions underlying the chi-square test were satisfied.

There were 65 first molar implants with fractured abutments belonging to patients with a GOA less than 120°, of which 40 (61.5%) were male and 25 (38.5%) were female. There were 19 first molar implants with fractured abutments belonging to patients with a GOA greater than 120°, of which 11 (58%) were male and 8 (42%) were female.

The hypothesis test showed an insignificant result (p >0.05; p = 0.7955) and the null hypothesis cannot therefore not be rejected. The conclusion from this is that the proportion of first molar implants with fractured abutments with a GOA of less than 120° is equal whether they belong to male or female patients.

### Results of abutment fracture rate in practice compared with total number of implants placed

From 2012 to the present, the implants were all restored as screw-retained on a titanium base as opposed to previously being cement-retained. The total number of implants placed and restored as screw-retained on a titanium base is and included in the audit is 17,207. There were 105 abutment fractures in the ten-year period of analysis, which gives an overall fractured abutment rate of 0.6%.

#### Abutment fracture rates in molar sites

Based on the findings, it was decided to look at some of the figures more deeply to see exactly which sites were more prone to abutment fracture. Of the 17,207 implants placed, 6,498 were molar implants (37.8% of total), and seeing as most of the titanium-base fractures were on molar implants, further study was conducted to collate these figures and calculate a percentage based on the teeth the implants were replacing.

Of these molars, 5,183 were first molars (79.8%) and 1,315 were second molars (20.2%). Moreover, 393 of these molars were abutments in fixed-fixed implant bridgework (first molars 289 [73.5%], second molars 104 [26.5%]) and thus excluded, as abutment fracture is almost non-existent in this scenario (bar one rogue case in the practice that was therefore omitted from the study as an outlier).

First molar-positioned implants that were charted as such within a full arch bridge (all-on-X) were also excluded (185) as again, abutment fracture is not a complication that has been seen in this treatment modality at the practice. This brings the total number of implants placed and restored as single molar units, screw-retained on a titanium base to 5,920 (4,709 first molars and 1,211 second molars). From this, 27% of the single implants placed at the practice are single-unit first molars.

#### Abutment fracture rates in first molar sites

Due to the higher proportion of abutment fractures belonging to the first molars, it was decided to then look at the differences between abutment fractures in relation to how many first molar implants had been placed. There was a total of 4,709 single-unit first molar placements restored as screw-retained crowns on a titanium base, and 87 of these suffered a fractured abutment, giving a percentage of 1.9%. Thus the abutment fracture rate for implants in the first molar region was 1.9%.

The four different molar sites were looked at individually to see if there was a difference in fracture rate between them (Table 3).

**Table 3:** Fracture abutment rates for each first molar site

**Site**	**Total restored**	**Number of # abutments**	**# rate/%**
16	960	16	1.7
46	1,293	24	1.9
26	956	15	1.6
36	1,500	32	2.1

### Results of comparison of the published failure rate from the literature review and the fracture rate in practice

The overall titanium base fracture rate in practice for the specified ten-year period for all implants placed and restored was 0.6% (105/17,207). The failure rate obtained from the literature based on the median fracture rate scores was 2.2%. Therefore, the overall fracture rate in practice according to the clinical audit is 1.6% lower than that reported in the literature.

A further standard was also set from four of the included studies that specifically looked at the Ankylos implant system. This gleaned an average percentage fracture rate of 1.8%, again showing the clinical audit fracture rate of 0.6% to be 1.2% lower.

Analysis of the audit data has revealed a pattern of the first molars being more implicated in the cases with fractured abutments. A further analysis of this has shown an overall fracture rate of all first molars placed in the audit time frame of 1.9%. Looking at percentage fracture rates over all single implants placed in the audit timeframe, the fracture rate for first molars was 0.5%. Therefore, 83% of all the fractured abutments recorded in the audit belonged to first molars. It is worth remembering that first molar implant placements formed 27% of the total implants placed overall in the timeframe analysed in the audit.

## Discussion

After reviewing the papers from the initial search, 26 were found to satisfy the inclusion criteria for more detailed analysis, which led to a resultant eight papers that could then be used to determine the published failure rate. Th average quality score was 17/24 which was felt to indicate that these were good-quality papers. These were all retrospective, clinical studies, bar one, which was a prospective clinical trial. However, this showed a 0% abutment fracture rate, so out of the eight studies, seven were used to determine the failure rate. It was then identified that four out of the eight studies focused on Ankylos implants specifically, thus proving to be of particular interest, as the intended clinical audit was to review the abutment fracture rates on this brand of implants.

The practice audit included records spanning more than ten years, which covered the average follow-up period determined from the literature search (eight years) and the clinical audit performed to ascertain the practice abutment fracture rate (ten years). The numbers of implants included varied, with a median of 694 implants analysed across the eight studies and a mean of 1,207 across the four Ankylos studies. The total number of Ankylos implants included in the clinical audit was 17,207, giving a sample size that is much higher than those of the included studies.

The overall abutment fracture rate from all eight studies (using the median) was 2.2% and for the four Ankylos studies, this was 1.8%. From the clinical audit, the overall abutment fracture rate was 0.6%, which is 1.6% less than the overall fracture rate and 1.2% less than the fracture rates in the published Ankylos studies.

One interesting observation is that the abutment fracture rate in the practice was less than that of the published literature, especially as the sample size analysed was far greater than those in the studies. The audit findings may help reduce the rate even further; the risk factors, which are now better understood, will be shared with the clinicians, and this could improve awareness, allowing the practice to have greater vigilance when consenting and planning cases.

### Differences in abutment fracture rate between men and women

The results from the clinical audit show that there is greater incidence of abutment fracture in men than women, which was found to be statistically significant (p <005; p = 0.0015). This finding correlates with other studies looking at the difference in occlusal forces generated by men and women,^14^^,^^18^ where Calderon *et al.*^18^ found that the average maximum occlusal force was statistically higher for men (587.2 N) than women (424.9 N). Five of the included studies used to determine the failure rate also agreed with this finding.^9^^,^^16^^,^^20^^,^^21^^,^^22^ Men are therefore at greater risk of abutment fracture than women.

### Age of patients with fractured abutments

The average age of the patients in the audit with fractured abutments was 61 years, which is nine years younger than the average age found in three of the included studies. This could be due to the predominant demographic treated at the practice, which is older than those of the included studies. Further analysis relating to the ages of patients treated in the ten-year period would need to be performed to know this for sure.

### Site

The molars contributed to the greatest amount of abutment fractures, with 92.5% of them belonging to the first and second molars and 7.5% of belonging to premolars. This correlates with the findings of others who all found the molar site to be more accountable for abutment fractures.^9^^,^^19^^,^^20^^,^^21^^,^^22^ By knowing molars are more prone to abutment fracture, the relative risk of this occurring can be deduced when planning cases. As will be discussed, the GOA measurement in combination with the molar site can help give patients some idea of their risk of abutment fracture during the consent process.

The first molars made up 83% of the total abutment fractures in the practice audit, yielding a first molar abutment fracture rate of 1.9% (of all single-unit screw-retained implants placed) and an overall fracture rate of 0.5% of all implants placed in the practice in the selected time frame. Additionally, 64% of these belonged to lower first molars and 36% to upper first molars, which at first indicated that lowers were more at risk than the uppers. However, on further analysis, there were no statistically significant differences between upper and lower first molars with respect to abutment fracture rate (p = 0.8).

The first molars are commonly missing, largely due to the fact they erupt early and have an associated high caries risk. Patients often seek to replace their missing first molars as mastication can be greatly impaired by their omission. Although there is no difference between the upper and lower first molars, this site is still at higher risk of abutment fracture.

From the statistical analysis of first molar abutment fracture between men and women, men are at greater risk than women (p = 0.0015). This could be an important consideration to make when consenting, planning and executing the treatment of male patients receiving molar site implants.

Other studies have found that abutment fracture is greater in implants with a wider diameter.^19^^,^^21^ Conversely, it has been found that implants with a narrower diameter are at greater risk of implant fracture which is a much more catastrophic consequence.^23^

### Period of functioning time in the mouth before abutment fracture

The average period of time the implant was functioning in the mouth before abutment fracture was 74.3 months (6.2 years). The practice currently encourages patients to return annually for a clinical and radiographic check of their implants. Part of the clinical examination involves checking the occlusion and making sure, when opposing a natural tooth, that the static and excursive occlusal contacts are not heavy or involved in an interference. If this is found to be the case, the occlusion is adjusted. It would be useful to perhaps be more aware of this being important around the 5-7-year post-completion mark, as this appears to be the time when abutment fracture is more prevalent.

### Retrieval

The audit showed that following abutment fracture, two patients did not return to have the apical portion retrieved and two were not able to be retrieved. This gives a retrieval success rate at the practice of 96%, which is possibly due to the expertise of the surgeons at the practice and their long experience of conducting the procedure. It is understood that the practice surgeons run internal courses to teach each other how to remove fractured abutments from implants. Also, the use of titanium bases eases the restoration, as if the crown itself is undamaged, a new titanium base can be rebonded to the crown (with or without a new digital intra-oral scan) and then fitted once back from the laboratory.

### Opposing natural tooth or implant?

The audit showed that 89% of implants with fractured abutments were also opposing a natural tooth, as opposed to 11% that were opposing an implant. Jin *et al.*^21^ also found that implant fracture was statistically significantly greater (p = 0.001), when opposing a natural tooth.

When an implant is restored against another implant, if both are installed together in occlusal harmony, the relationship should remain static. An opposing tooth with a periodontal ligament can of course move and in time, if the occlusion is not checked and adjusted, occlusal interferences can be introduced to the implant restoration, which could mean componentry is at risk of fracture. This is something that can be checked during patient reviews, helping to ensure the occlusion is in intercuspal position and laterotrusion is not abnormally heavy, and the occlusion is adjusted as needed to help prevent any kinds of fracture within the implant system.

### Terminal tooth/bound

Analysis has shown that an implant presenting with a fractured abutment was more likely to be bound by another tooth/implant, rather than being the most terminal unit in the arch. It is not clear as to why this should be. To know exactly how significant these variables are in terms of overall abutment fracture risk, further data on the implants without fractured abutments would need to be collected and analysed. It does, however, give another relative risk factor for the practice to be aware of when consenting and planning such cases.

### Measurement of gonial angle from orthopantomograms

The audit conducted showed that 77.4% of first molar fractured abutments belonged to patients with a GOA less than 120°, which agrees with the findings of others^14^^,^^16^^,^^24^ (that patients with a gonial angle of less than 120° usually exert a greater occlusal force in the molar regions). However, the results of the audit found no difference in first molar abutment rate fracture in patients with a GOA of less than 120° between men and women.

An area of focus in any follow-up study might be the GOA for all molar implant cases. It would be interesting to compare cases with and without abutment fracture, to gain some understanding of the fracture risk in relationship to GOAs.

By looking at the data collected from the clinical audit, it would be reasonable to suggest that the following factors may be a potential causative factor in implant abutment fracture:

- Men at more risk than women
- Patient age ~60 years
- First molar site
- Implants that have been functioning in the mouth for approximately six years
- Implants opposing a natural tooth
- Implants with another tooth/implant distal to themselves
- Patients with a GOA <120° as measured on their OPG.

Further investigation and analyses are required to delve deeper into these variables and see exactly how important they are with respect to abutment fracture risk. It would be advisable to be more aware of the risk for male patients receiving first molar implants with a GOA of <120°. The other variables require further investigation to know more precisely how important they are.

## Conclusion

The information obtained from the literature for the fractured abutment rate in practice was 2.2%. The four studies looking at Ankylos abutment fracture gleaned an average rate of 1.8%. The overall fractured abutment rate determined from the clinical audit for the practice for the defined ten-year period was 0.6%. This was further analysed to calculate the fracture rate for the first molars which accounted for 84% of the total fractured abutments in practice. This yielded a first molar abutment fracture rate of 1.9% (of all single-unit screw-retained implants placed) and an overall fracture rate of 0.5% of all implants placed in the practice in the defined timeframe.

This concludes that the overall abutment fracture rate is less than that determined from the literature.

Looking to the future, an attempt to bring this figure down ever further should be made and this can be done by realising that men receiving first molar implants with a GOA of less than 120° are statistically more at risk of abutment fracture. Other variables, such as opposing structure, period of time that the implant has been in function and whether the implant is the most distal tooth in the arch or not, require further investigation and analysis before these can be deemed as statistically significant risk factors.

## Data Availability

Data can be made available subject to patient confidentiality requirements.
